# Knockdown of KIAA1199 attenuates growth and metastasis of hepatocellular carcinoma

**DOI:** 10.1038/s41420-018-0099-5

**Published:** 2018-11-12

**Authors:** Jingmei Liu, Ping Han, Jin Gong, Yunwu Wang, Bingxin Chen, Jiazhi Liao, Dean Tian

**Affiliations:** Department of Gastroenterology, Tongji Hospital, Tongji Medical College, Huazhong University of Science and Technology, Wuhan, China

## Abstract

Accumulating evidence indicates that KIAA1199 plays a vital role in tumor progression. However, the role of KIAA1199 in hepatocellular carcinoma (HCC) still remains unknown. In this study, we found that KIAA1199 was upregulated in human HCC tissues and in highly metastatic HCC cell lines. Furthermore, the expression of KIAA1199 was significantly correlated with tumor size and metastasis in HCC. Knockdown of KIAA1199 inhibited cell proliferation and migration in vitro, and suppressed tumorigenicity and lung metastasis in vivo. In addition, silencing of KIAA1199 induced G1 phase arrest by reducing cyclinD1 expression. Moreover, KIAA1199 knockdown induced apoptosis by activating endoplasmic reticulum (ER) stress, which was based on the upregulation of ER stress markers, activating transcription factor 4 (ATF4) and CAAT/enhancer-binding protein homologous protein (CHOP). In conclusion, our data demonstrated that KIAA1199 knockdown inhibited the growth and metastasis of HCC.

## Introduction

Hepatocellular carcinoma (HCC) is the third leading cause of cancer-related mortality worldwide^1^. More than 90% of cancer-related deaths are the consequence of the tumor cells escaped from primary tumor to form metastases^2^. Although genetic and epigenetic alterations are well characterized in HCC, the molecular pathogenesis still remains largely unknown^3^. Thus, additional studies must be carried out to gain a better understanding of HCC progression in order to develop novel therapeutic targets.

KIAA1199 was first identified as an inner ear-specific protein, which is located on chromosome band 15q25.1^4^. It has been reported that KIAA1199 is upregulated in many types of tumors, such as colorectal cancer, gastric cancer, and breast cancer^5–7^. Moreover, high levels of KIAA1199 were correlated with worse five-year survival outcomes in colon cancer^8^ and lymph node metastasis in gastric cancer^7^. Evensen et al. found that KIAA1199-mediated cell migration relies on ER Ca^2+^ leakage, followed by the protein kinase C-α isoform (PKCα) activation^9^. These findings are consistent with a recent report, which showed that repression of KIAA1199 decreased the proliferation and invasion of colon cancer cells by modulating Ca^2+^ signaling^10^. Together, these results strongly suggest a significant role of KIAA1199 in tumor progression through diverse mechanisms. However, the exact function of KIAA1199 in HCC has not been investigated yet.

In the present study, we demonstrated that KIAA1199 was upregulated in HCC tissues, and the expression of KIAA1199 was positively correlated with the metastatic potentials of HCC cells. Furthermore, we also confirmed that KIAA1199 knockdown inhibited the growth and metastasis of HCC by both in vitro and in vivo assays. Mechanistic studies revealed that KIAA1199 knockdown induced cell apoptosis by activating endoplasmic reticulum (ER) stress. Moreover, these findings provided novel mechanistic insights into the function of KIAA1199.

## Results

### Expression and clinicopathologic significance of KIAA1199 in HCC

**Fig. 1:**
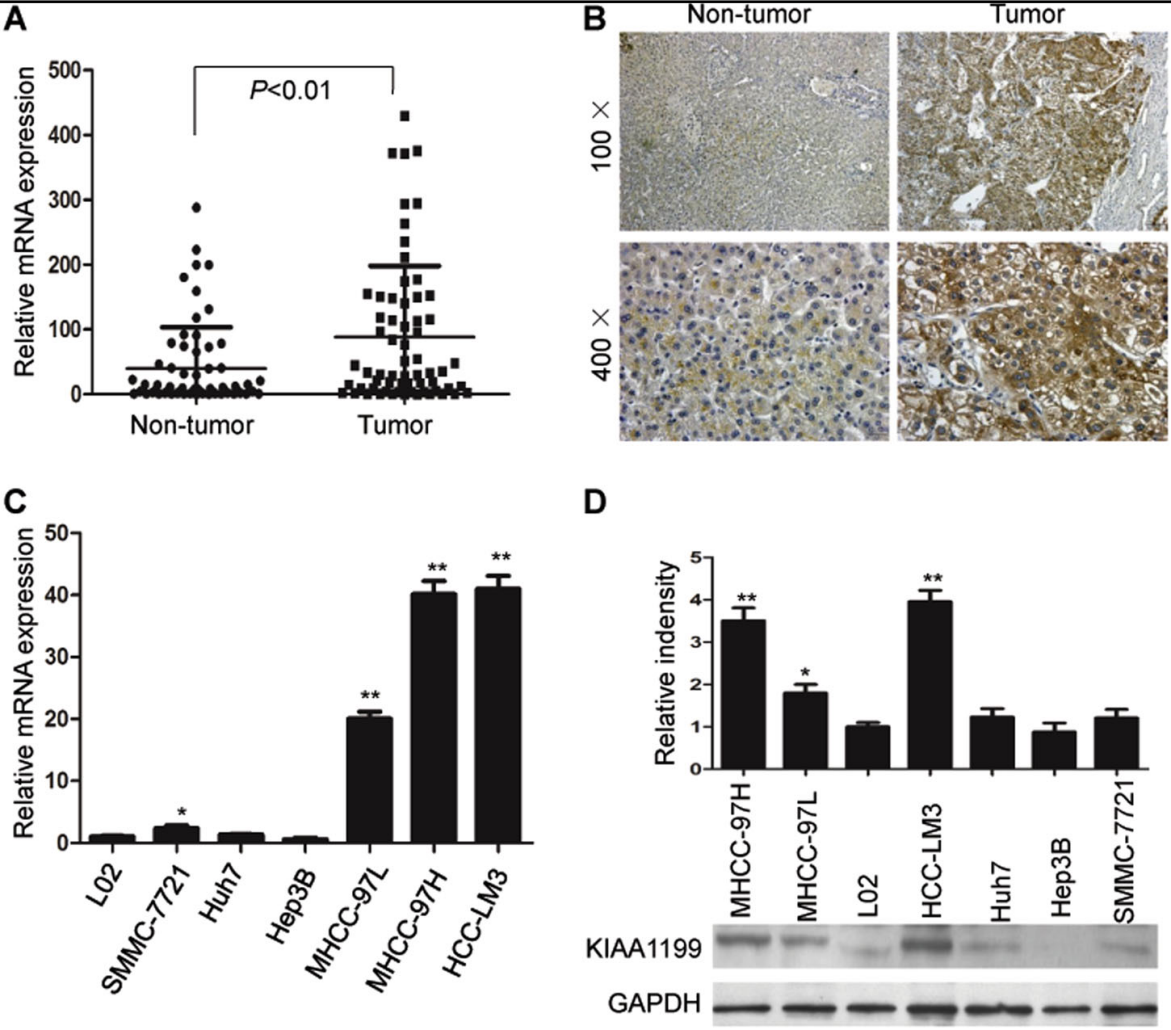
KIAA1199 is upregulated in HCC tissues and highly invasion HCC cells. **a** RT-qPCR analysis of KIAA1199 expression in 64 pairs of HCC tissues. **b** Representative immunohistochemical staining showed the expression of KIAA1199 in liver cancer tissues and the corresponding pericarcinoma tissues. **c** RT-qPCR and **d** western blot analysis of KIAA1199 expression in the normal liver cell line L02 and six HCC cell lines (MHCC-97H, MHCC-97L, HCC-LM3, SMMC-7721, Huh7, and Hep 3B). GADPH was used as the loading control. Data are represented as the mean ± SD. *P < 0.05, **P *<* 0.01

To explore the effect of KIAA1199 on HCC, RT-qPCR and immunohistochemistry was performed to determine the expression of KIAA1199 in 64 pairs of HCC samples. Compared with the corresponding pericarcinoma tissues, KIAA1199 was significantly upregulated in 47 pairs of HCC tissues (73.44%) (Fig. 1a), and staining of KIAA1199 in HCC samples showed positivity mostly in the cytoplasm (Fig. 1b). Further analysis of the clinicopathological characteristics in HCC samples showed that KIAA1199 overexpression positively correlated with tumor size (*P* *=* 0.043) and metastasis (*P* *=* 0.023), but no significant differences were observed with respect to age, sex, tumor number, and the alpha-fetoprotein (AFP) level (Table 1). In addition, we also analyzed the expression of KIAA1199 in the normal liver cell line L02 and six HCC cell lines. In accord with the results from tissues, we found that KIAA1199 was overexpressed in highly invasive HCC cell lines and hardly any in the normal liver cell line (Fig. 1c, d). These results indicated that KIAA1199 played an important role in HCC progression.

**Table 1:** KIAA1199 expression and clinicopathological factors

Variable		KIAA1199		*P* value
		High expression (*n* = 47)	Low expression (*n* = 17)	
Sex	Male	43	14	0.561
	Female	4	3	
Age (year)	≤45	16	6	0.924
	>45	31	11	
HBsAg	Positive	35	12	0.748
	Negative	12	5	
AFP (ng/ml)	≤400	14	7	0.392
	>400	33	10	
Tumor diameter	≤5	10	8	0.043*
(cm)	>5	37	9	
Tumor number	Single	38	11	0.182
	Multi	9	6	
Metastasis	Yes	34	7	0.023*
	No	13	10	

KIAA1199 high (low) expression (whose relative expression of HCC tissues was higher (lower) than that of corresponding pericarcinoma tissues according to the results of PCR). *AFP* alpha-fetoprotein

*Indicates statistical significance

### KIAA1199 knockdown inhibits HCC cell proliferation and migration

**Fig. 2:**
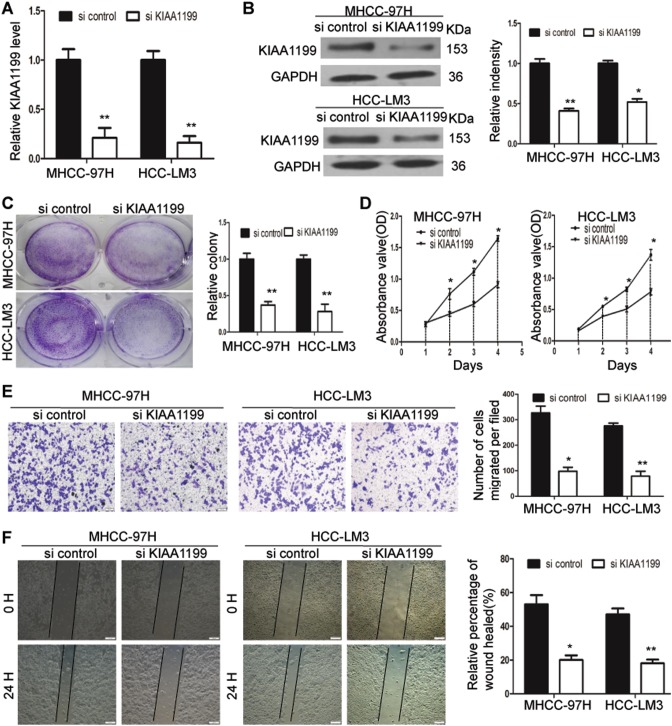
KIAA1199 knockdown inhibits HCC cell proliferation and migration. **a**, **b** The expression of KIAA1199 in MHCC-97H and HCC-LM3 cells transfected with siRNAs (si control and si KIAA1199) were determined by RT-qPCR (**a**) and western blot (**b**). GADPH was used as the loading control. **c** Cell colony formation was detected. Representative micrographs (left) and quantification (right) of crystal violet-stained cell colonies in MHCC-97H and HCC-LM3 cells. **d** The cell proliferation of MHCC-97H and HCC-LM3 cells transfected with siRNAs (si KIAA1199 or si control) were determined by CCK-8 assay. **e** Cell migration in MHCC-97H and HCC-LM3 cells were analyzed by transwell assay. Scale bars = 100 μm. **f** Wound healing assay showed, compared with the control group, that wound closure was delayed in MHCC-97H and HCC-LM3 cells transfected with siKIAA1199 at 24 h. Scale bars = 100 μm. Each bar represents the mean ± SD of three separate experiments. *P < 0.05, **P *<* 0.01

To investigate the functional roles of KIAA1199 in HCC, KIAA1199 expression in metastatic HCC cell lines (HCC-LM3 and MHCC-97H) was suppressed by RNA interference (RNAi). Successful knockdown of KIAA1199 was illustrated by RT-qPCR and western blotting (Fig. 2a, b). Initially, the capacity of colony formation was evaluated in HCC-LM3 and MHCC-97H cells that were transfected with si KIAA1199 or si control. Compared with the si control-transfected cells, cells transfected with si KIAA1199 had much fewer and smaller colonies (Fig. 2c). Next, the effect of KIAA1199 on cell proliferation was assessed by CCK-8 assays. The results demonstrated that HCC-LM3 and MHCC-97H cells transfected with siKIAA1199 showed a lower proliferation rate than the control cells (Fig. 2d). Furthermore, transwell and wound healing assays revealed that silencing of KIAA1199 caused a suppression of cell migration in HCC-LM3 and MHCC-97H cells (Fig. 2e, f). Therefore, KIAA1199 knockdown inhibited HCC cell proliferation and migration.

### KIAA1199 knockdown suppresses the growth and metastasis of HCC in vivo

**Fig. 3:**
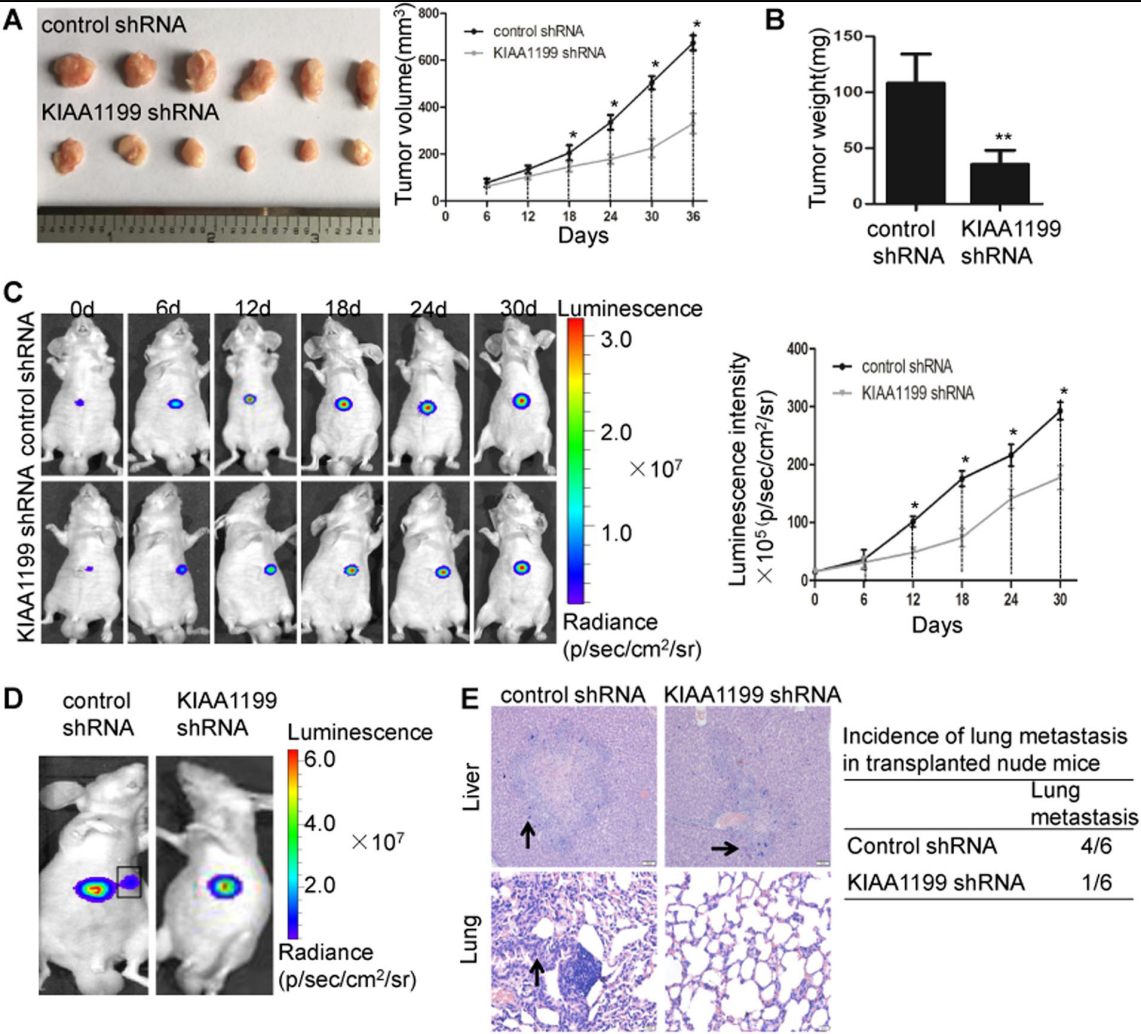
KIAA1199 knockdown suppresses the growth and metastasis of HCC in vivo. **a** Images (left) and the growth curves (right) of tumors in each group (*n* = 6); the tumor volumes were estimated using calipers. **b** Tumor weight in each group was measured on day 36. Each bar represents the mean ± SD of six mice per group. **c**, **d** Representative bioluminescence (BLI) images showed primary liver tumors (**c**) and lung metastases (**d**) in orthotopic xenograft models at day 60. **e** Representative H&E staining of liver and lung in orthotopic xenograft models and the incidence of lung metastasis in each group of nude mice were shown. Original magnification: ×100. Black arrows indicate tumors in the liver and lung. **P < 0.01

Having observed that KIAA1199 played a critical role in promoting HCC cells proliferation and migration in vitro, we evaluate the function of KIAA1199 on tumor growth and metastasis by subcutaneous xenograft models and orthotopic xenograft models, respectively. HCC-LM3-shRNA and HCC-LM3-shKIAA1199 cells were subcutaneously injected into nude mice, and tumor growth was assessed. Compared with the HCC-LM3-shRNA group, the sizes and weight of tumors in HCC-LM3-shKIAA1199 group was decreased (Fig. 3a, b). In orthotopic xenograft models, tumor development was monitored by bioluminescent (BLI) imaging. BLI imaging revealed that the growth of tumors in HCC-LM3-shKIAA1199 group was slower than the HCC-LM3-sh control group (Fig. 3c). Moreover, BLI imaging and H&E staining revealed significant lung metastasis in the HCC-LM3-sh control group, while in the HCC-LM3-shKIAA1199 group, little to no lung metastasis was detected (66.7% versus 16.7%) (Fig. 3d, e). Collectively, these results suggested that reduction of KIAA1199 inhibited HCC growth and metastasis in vivo.

### KIAA1199 knockdown induces G1 arrest by reducing cyclinD1

**Fig. 4:**
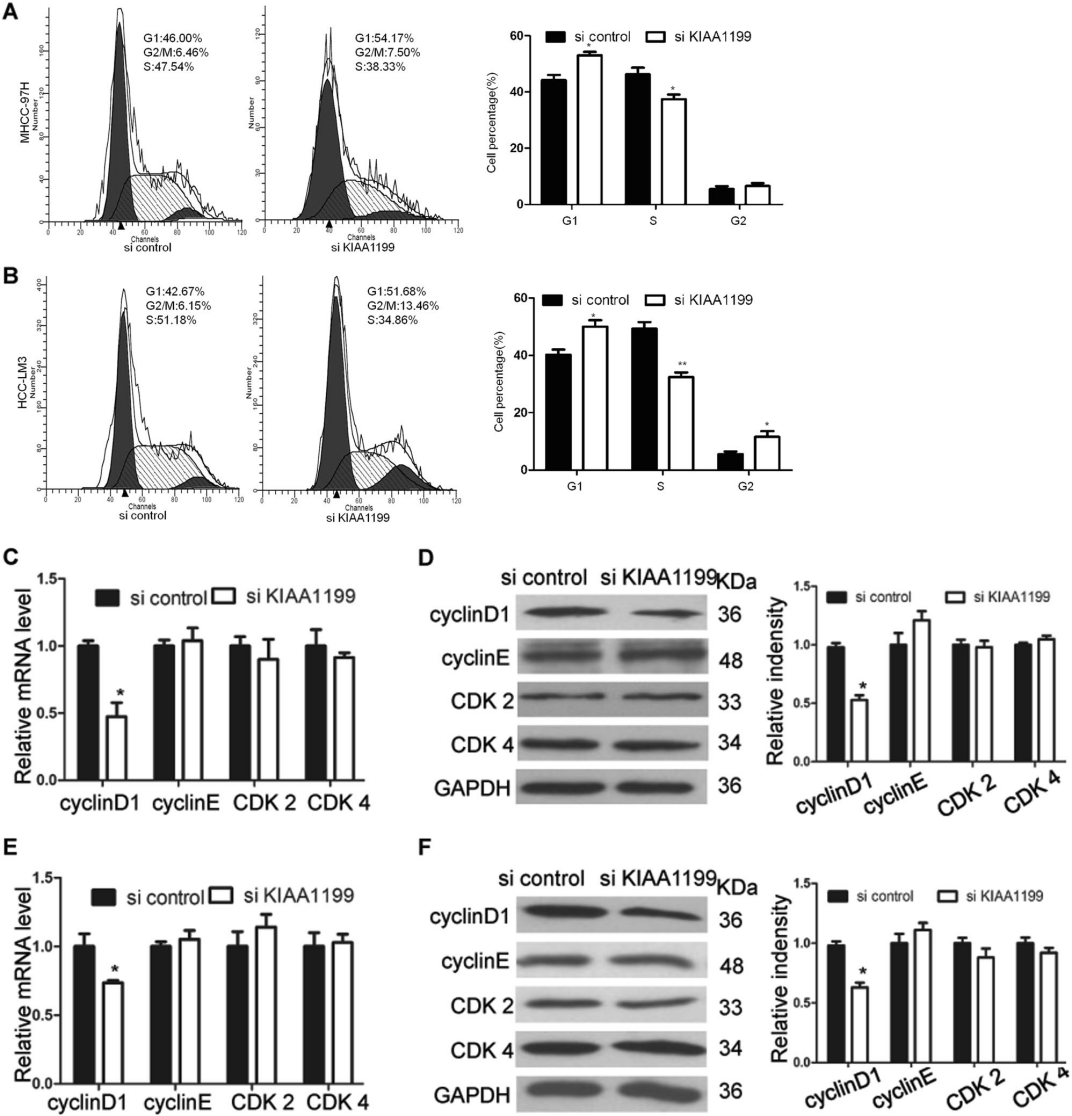
KIAA1199 knockdown induces G1 arrest by reducing cyclinD1. **a**, **b** The cell cycle distribution in MHCC-97H and HCC-LM3 cells transfected with siRNAs (si control and si KIAA1199) were analyzed by flow cytometry. **c**, **d** The levels of cyclinD1, cyclinE, CDK2, and CDK4 in MHCC-97H cells were determined by RT-qPCR (**c**) and western blot (**d**). **e**, **f** The levels of cyclinD1, cyclinE, CDK2, and CDK4 in HCC-LM3 cells were determined by RT-qPCR (**e**) and western blot (**f**). GAPDH was used as the loading control. Each bar represents the mean ± SD of three separate experiments. *P < 0.05, **P < 0.01

Silencing of KIAA1199 suppressed the growth of HCC cells. To elaborate the mechanism of this inhibitor effect, we evaluated the cell cycle distribution of HCC cells by flow cytometry. Compared with the control cells, MHCC-97H and HCC-LM3 cells transfected with si KIAA1199 showed a significant decrease of the percentage of cells in the S phase, and cell cycle was significantly blocked at the G1 checkpoint (Fig. 4a, b). As KIAA1199 knockdown caused cell cycle arrest in G1 phase, we investigated whether the expression of several key factors regulating G1/S cell cycle transition were affected. The results demonstrated that reduction of KIAA1199 decreased the level of cyclinD1, while the expression of cyclinE, cyclin-dependent kinases 2 (CDK2), and cyclin-dependent kinases 4 (CDK4) were not observably affected in both MHCC-97H (Fig. 4c, d) and HCC-LM3 cells (Fig. 4e, f). These data indicated that KIAA1199 knockdown induced cell cycle arrest by reducing cyclinD1 gene expression.

### KIAA1199 knockdown induces ER stress-mediated apoptosis

**Fig. 5:**
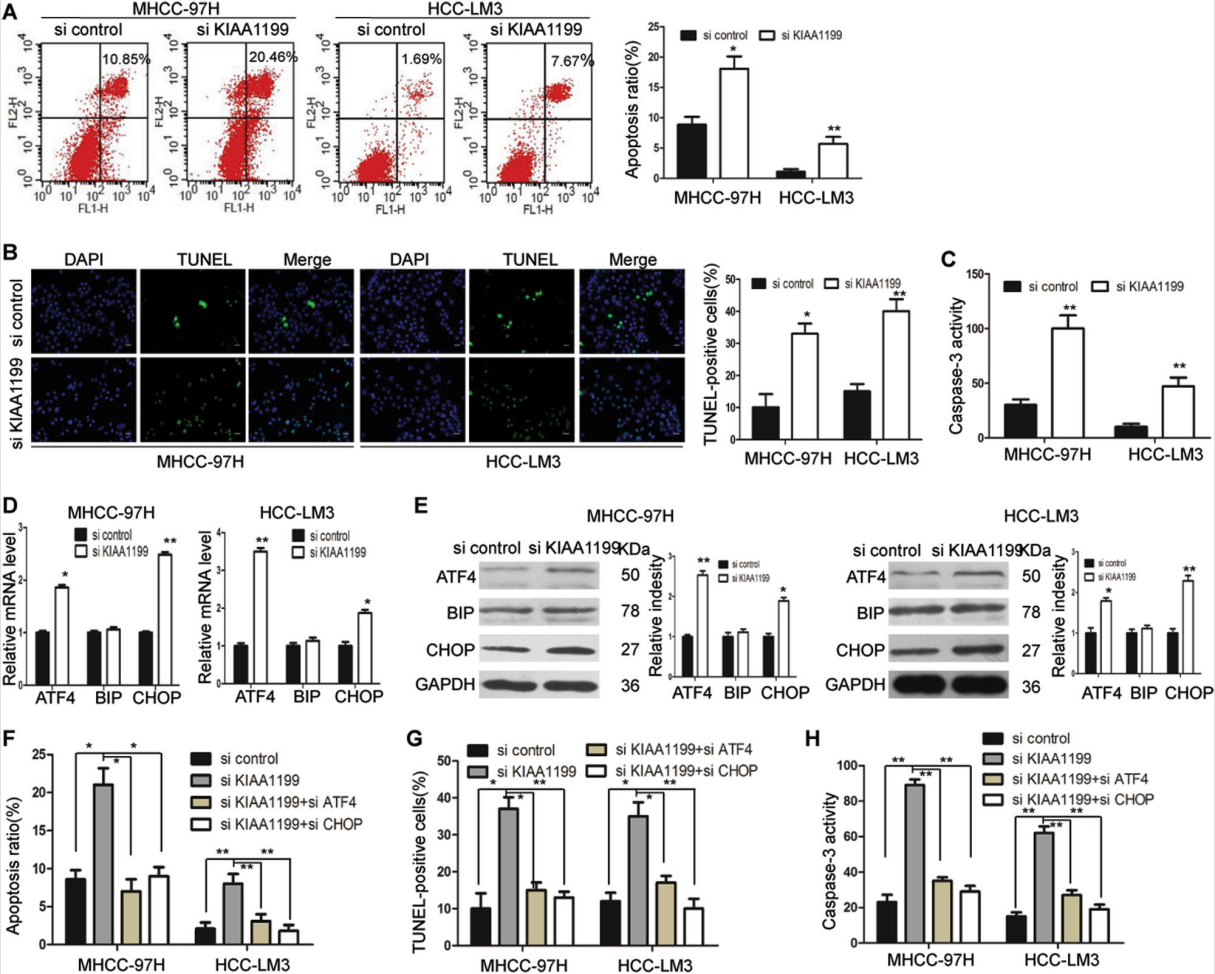
KIAA1199 knockdown induces ER stress-mediated apoptosis. **a** FACS analysis of treated MHCC-97H and HCC-LM3 cells labeled with Annexin V-FITC and propidium iodide (PI) as markers for apoptosis. **b** Representative images and quantitative analysis of TUNEL assay in MHCC-97H and HCC-LM3 cells transfected with siRNAs (si control and si KIAA1199) were presented. Scale bars = 100 μm. **c** Caspase-3 activity of MHCC-97H and HCC-LM3 cells transfected with siRNAs (si control and si KIAA1199) were detected. **d** RT-qPCR and **e** Western blot analysis of ER stress-related protein expression in MHCC-97H and HCC-LM3 cells transfected with siRNAs (si control and si KIAA1199), including CHOP, ATF4, and Grp78(BIP). GAPDH was used as the loading control. **f** Quantitative analysis of cell apoptosis, **g** TUNEL assay, and **h** caspase-3 activity in MHCC-97H and HCC-LM3 cells transfected with si control, siKIAA1199, siKIAA1199+siATF4, and siKIAA1199+siCHOP. Each bar represents the mean ± SD of three separate experiments. *P < 0.05, **P < 0.01

Next, we explored the effect of KIAA1199 on cell apoptosis. Analysis of apoptosis by flow cytometry demonstrated that repression of KIAA1199 significantly increased the percentage of apoptotic cells in both HCC-LM3 and MHCC-97H cells (Fig. 5a). We also detected the effect of KIAA1199 knockdown on cell apoptosis in HCC-LM3 and MHCC-97H cells by TUNEL assay and caspase-3 activity assay. Compared with the cells transfected with si control, si KIAA1199 treatment increased the number of TUNEL-positive cells (Fig. 5b). Expectedly caspase-3 activity was also induced in MHCC-97H and HCC-LM3 cells after KIAA1199 silencing (Fig. 5c). Accumulating evidences indicate that persistent and excessive ER stress can cause an apoptotic response^11^. Activating transcription factor 4 (ATF4) and CAAT⁄enhancer-binding protein homologous protein (CHOP) are the key signal molecules that lead to ER stress-mediated apoptosis^12^. To determine whether ER stress played a role in KIAA1199 knockdown-induced apoptosis, the levels of ER stress-associated markers were detected in HCC cells transfected with siRNAs (si control or si KIAA1199). The results showed that silencing of KIAA1199 significantly induced the expression of CHOP and ATF4, but had no impact on the expression of glucose-regulated protein, 78 kDa (Grp-78, BiP), which has a positive regulatory role in the prevention of apoptosis (Fig. 5d, e). Our data also demonstrated that transfection with ATF4 siRNA and CHOP siRNA significantly attenuated cell apoptosis induced by KIAA1199 knockdown (Fig. 5f). Moreover, the TUNEL-positive cells and the caspase-3 activity increased by KIAA1199 knockdown were decreased by siATF4 and siCHOP (Fig. 5g, h). Taken together, KIAA1199 knockdown induced ER stress-mediated apoptosis in HCC cells.

## Discussion

Although upregulation of KIAA1199 has been observed in various types of human cancer, such as breast cancer, gastric cancer, and colon cancer^5,7,13,14^, the role of KIAA1199 in HCC still remains unknown. In this study, for the first time, we found that KIAA1199 was significantly overexpressed in HCC tissues, especially in those with lager tumor size and metastasis. Moreover, KIAA1199 expression was positively correlated with the metastatic potential of HCC cells. Knockdown of KIAA1199 attenuated HCC growth and metastasis, which was supported by both in vitro and in vivo experiments. Our findings reveal that KIAA1199 may serve as a novel oncogene in the pathogenesis and progression of HCC.

It is well-known that uncontrolled cell proliferation is the key mechanism for the progression of human malignancies^15^. Birkenkamp–Demtroder et al. reported that repression of KIAA1199 decreased the proliferation of colon cancer cell line SW480^5^. Our results indicated that KIAA1199 knockdown significantly inhibited HCC cell proliferation. This was further supported by the result that silencing of KIAA1199 inhibited the growth of xenograft tumors in mice. To explore the underlying molecular mechanism, we focused on the cell cycle. Interestingly, suppression of KIAA1199 induced cell cycle arrest at G1 phase, and the expression of cyclinD1, a crucial regulator in cell cycle arrest, was downregulated by KIAA1199 knockdown in HCC cells.

An essential hallmark of cancer cells is the ability to evade apoptosis^16^. As we know, moderate ER stress can act as a protective mechanism, however, prolonged and excessive ER stress eventually leads to apoptosis^17,18^. Our data showed that KIAA1199 knockdown induced apoptosis in HCC cells. Furthermore, we also confirmed that reduction of KIAA1199 induced ER stress, as indicated by the upregulation of ER stress-related genes. CHOP and Grp78(BiP) are two important effectors in ER stress^19^. CHOP has been reported to be a crucial ER stress responsive factor that executes apoptosis, which is also a downstream target of ATF4 pathway. Grp78(BiP), an ER stress chaperone molecule, is upregulated in ER stress^12^. Evensen et al. showed that KIAA1199–BiP interaction is a specific cellular event required for ER retention and cell migration in breast cancer^9^. However, we found that reduction of KIAA1199 increased the levels of ATF4 and CHOP, but exerted no effect on BIP expression in HCC cells. Moreover, ATF4 siRNA and CHOP siRNA attenuated KIAA1199 knockdown-induced apoptosis. This study provides important evidence that KIAA1199 knockdown induced ER stress-mediated apoptosis in HCC cells. The possibility of involvement of other factors in apoptosis induced by KIAA1199 knockdown could not be excluded.

In conclusion, our present study suggests that KIAA1199 is involved in the development and progression of HCC. KIAA1199 knockdown inhibited cell proliferation and migration in vitro and attenuated tumor growth and metastasis in vivo. All of these results provide a wider perspective on HCC treatment.

## Materials and methods

### HCC samples and cell lines

The 64 paired tissue specimens were obtained from HCC patients (Department of Surgery, Tongji Hospital of Tongji Medical College, Huazhong University of Science and Technology, between 2011 and 2014) by way of surgery after getting their consent. The cell lines HCC-LM3, MHCC-97H, MHCC-97L, SMMC-7721, Huh7, Hep3B, and L02 (Institute of liver diseases, Tongji Hospital of Tongji Medical College, Huazhong University of Science and Technology, Wuhan, Hubei, China) were cultured in DMEM medium containing 10% fetal calf serum (Invitrogen Gibco, Carlsbad, CA, USA) and incubated in a 5% CO_2_ incubator at 37 °C. All human and animal studies were performed according to the guidelines of the Ethics Committee of the Tongji Hospital and approved in accordance with the ethical standards of the World Medical Association Declaration of Helsinki.

### RNA extraction and real-time RT-PCR

Total RNA was extracted using TRIzol reagent (Invitrogen, Carlsbad, CA, USA). Reverse-transcribed complementary DNA was synthesized using the PrimeScript RT reagent kit (TaKaRa, Otsu, Japan). Real-time polymerase chain reaction was performed using SYBR Premix ExTaq (TaKaRa, Otsu, Japan) on an ABI StepOne Real-Time PCR System (Applied Biosystem, Carlsbad, CA, USA). The sequences of the primers used for PCR were listed in Supplemental Table 1.

### RNAi and establishment of overexpressing cells

RNAi and establishment of stable expressing cells were performed as described previously^20^. siRNAs were synthesized by RiboBio company (Guangzhou, China) and plasmids were synthesized by Vigenebio Company (Shandong, China), and then transfected into cells using lipofectamine 2000 (Invitrogen) according to the manufacturer’s instructions. The sequence of siKIAA1199 (or shKIAA1199) was designed as follows: GATCCTTACTATGGTCTGA. ATF4 siRNA sequence was designed as follows: AGGAGCAAAACAAGACAGCATTTT. CHOP siRNA sequence was designed as follows: GAGCUCUGAUUGACCGAAUTT.

### Wound healing and transwell assays

Wound healing and transwell assays were performed as described previously^20^.

### CCK-8, colony formation, and caspase-3 activity assays

CCK-8, colony formation, and caspase-3 activity assays were performed as described previously^21^.

### Cell cycle and apoptosis analysis

Cell cycle analysis kit and Annexin V-FITC apoptosis kit were purchased from BD Pharmingen (San Diego, CA, USA). For cell cycle analysis, the cells were harvested after treatment, fixed with ice-cold 70% ethanol solution, hydrolyzed with RNaseA, and stained with propidium iodide (PI) for 20 min. For apoptosis analysis, the cells were harvested after treatment, washed twice with PBS, and resuspended in 1×binding buffer. Annexin V-FITC and PI were added to the cell preparations and then incubated for 15 min in the dark. Cell cycle and apoptosis analysis were performed by FACS Calibur flow cytometer (Becton Dickinson, San Diego, CA).

### TUNEL assay

The apoptosis assay was measured by DeadEnd™ Fluorometric TUNEL System (Promega, Madison, WI). After 48 h of transfection with siRNAs or plasmids, TUNEL assay was performed according to the manufacturer’s instructions. Fluorescent images were captured using a Zeiss fluorescence microscope. For quantitative results, the TUNEL-positive cells were counted in five random area images for each sample by ImageJ.

### Western blotting and immunohistochemistry

For western blotting, proteins were separated on SDS-PAGE and transferred to nitrocellulose membrane (Bio-Rad). The membrane was blocked with 5% non-fat milk and incubated with the corresponding antibodies. Informations of the antibodies are listed in the Supplementary Table 2.

For immunohistochemistry, paraffin-embedded tissues were cut into 4-μm-thick consecutive sections. Antigen retrieval was performed following the standard procedure. Sections were cooled and immersed in a 0.3% hydrogen peroxide solution to block the endogenous peroxidase activity. Non-specific labeling was blocked with 5% non-fat milk, and then incubated with corresponding antibody, developed by peroxidase-conjugated streptavidin and DAB, and counterstained by hematoxylin.

### In vivo tumor growth and metastasis assays

For subcutaneous xenograft models, 5 × 10^6^ LM3-shRNA or LM3-shKIAA1199 cells in 0.1 ml PBS were injected into nude mice subcutaneously. The length (*L*) and width (*W*) of the tumors were measured with digital vernier calipers. Tumor volume (*TV*) was determined according to the formula: *TV* = (*L* × *W*^2^)⁄2. For orthotopic xenograft models, 1 × 10^6^ cells in 0.2 ml PBS were injected into the subcutaneous region of nude mice. Subcutaneous tumors were harvested once reached about 10 mm^3^, and then cut into 1.0-mm^3^ pieces. One piece of tumor was implanted into the left liver lobes of the nude mice. Mice were sacrificed on day 60. Liver and lung tissues were resected and fixed with 4% paraformaldehyde, and then stained with H&E.

### Statistic analyses

All experiments in vitro were performed in triplicate unless specified. Results are represented as the mean ± SD. KIAA1199 expression was compared with demographic and biological parameters by *χ*^2^ test. Statistical analysis was performed using Student’s *t* test. The *P* values < 0.05 were considered significant.

## Electronic supplementary material

Table S1. Primer sequences for RT-PCR

Table S2. Primary Antibodies for western blot

Declaration of contributions to article
