# Supplementary material for: Knockdown of KIAA1199 attenuates growth and metastasis of hepatocellular carcinoma
Source: Cell Death Discov. 2018 Nov 12;4:102. doi: 10.1038/s41420-018-0099-5 (PMC6232158; doi:10.1038/s41420-018-0099-5)
Supplement: Supplementary file 1 — Table S1. Primer sequences for RT-PCR [file 41420_2018_99_MOESM1_ESM.doc]

**Table S1. Primer sequences for RT-**PCR

| **Gene** | **Forward (5' -> 3')** | **Reverse (5' -> 3')** |
| --- | --- | --- |
| KIAA1199 | CACGGTCTATTCCATCCACATC | GGTTCGCAAAACAATCGGCT |
| Cyclin D1 | GCGTACCCCGATGCCAACCT | CCTCGCAGACCTCCAGCATCC |
| Cyclin E | CAGGGGCGTCGCTGATGAAGA | TGCTCGGGCTTTGTCCAGCAA |
| CDK2 | CCAGGAGTTACTTCTATGCCTGA | TTCATCCAGGGGAGGTACAAC |
| CDK4 | ATGGCTACCTCTCGATATGAGC | CATTGGGGACTCTCACACTCT |
| ATF4 | CCCTTCACCTTCTTACAACCTC | TGCCCAGCTCTAAACTAAAGGA |
| CHOP | GGAAACAGAGTGGTCATTCCC | CTGCTTGAGCCGTTCATTCTC |
| BiP | CATCACGCCGTCCTATGTCG | CGTCAAAGACCGTGTTCTCG |
| GAPDH | TCATTGACCTCAACTACATGGTTT | GAAGATGGTGATGGGATTTC |
