# Supplementary material for: Knockdown of KIAA1199 attenuates growth and metastasis of hepatocellular carcinoma
Source: Cell Death Discov. 2018 Nov 12;4:102. doi: 10.1038/s41420-018-0099-5 (PMC6232158; doi:10.1038/s41420-018-0099-5)
Supplement: Supplementary file 2 — Table S2. Primary Antibodies for western blot [file 41420_2018_99_MOESM2_ESM.doc]

**Table S2. Primary Antibodies for Western blot**

| **Protein** | **Concentration for western blot** | **Company** |
| --- | --- | --- |
| KIAA1199 | 1:1000 | Abnova |
| E-cadherin | 1:1000 | Proteintech |
| Vimentin | 1:1000 | Proteintech |
| Slug | 1:1000 | Proteintech |
| Twist1 | 1:1000 | Proteintech |
| Cyclin D1 | 1:1000 | Cell Signaling Technology |
| Cyclin E | 1:1000 | Cell Signaling Technology |
| CDK2 | 1:1000 | Cell Signaling Technology |
| CDK4 | 1:1000 | Cell Signaling Technology |
| ATF4 | 1:500 | Proteintech |
| CHOP | 1:500 | Proteintech |
| BiP | 1:200 | Proteintech |
| GAPDH | 1:10000 | Promoter |
