# Supplementary material for: Knockdown of KIAA1199 attenuates growth and metastasis of hepatocellular carcinoma
Source: Cell Death Discov. 2018 Nov 12;4:102. doi: 10.1038/s41420-018-0099-5 (PMC6232158; doi:10.1038/s41420-018-0099-5)
Supplement: Supplementary file 3 — Declaration of contributions to article [file 41420_2018_99_MOESM3_ESM.pdf]

DECLARATION OF CONTRIBUTIONS TO ARTICLE

ADMC

Manuscript Number: CDDISCOVERY-00452R

Journal Name: *Cell Death Discovery* (the 'Journal')

Proposed Title of the Contribution: Knockdown of KIAA1199 attenuates growth and metastasis of hepatocellular carcinoma (the 'Contribution)

Author(s): Jingmei Liu, Ping Han, Jin Gong, Yunwu Wang, Bingxin Chen, Jiazhi Liao, Dean Tian (the 'Authors')

For all *CDDis*covery articles, each person named as an author in the published version must be able to show he or she has contributed substantially to the article.

Authorship credit should be based on 1) substantial contributions to conception and design, acquisition of data, or analysis and interpretation of data; 2) drafting the article or revising it critically for important intellectual content; and 3) final approval of the version to be published. Authors should meet conditions 1, 2 and 3.

Any person who cannot be shown to have made a substantial contribution to the article cannot be listed as an author in the final version. The name of any person who is deemed to have made a minor contribution can, however, appear in the Acknowledgments section of the article.

Please complete the table below to indicate the contributions of all named authors to the manuscript.

Author Full Name:	Specification of Contribution to the Manuscript:
Jingmei Liu	She designed experiments and helped write the manuscript
Ping Han	He did the animal experiments
Jin Gong	She did the cell proliferation and apoptosis assay(including caspase-3 assay)
Yunwu Wang	He did the cell migration assay,Tunel assay
Bingxin Chen	She did the PCR,western blot and IHC assay
Jiazhi Liao	He helped write the manuscript
Dean Tian	He analysed the data and helped write the manuscript

Please complete the table below to indicate the contributions of all named authors to the figures.

Figure 1:

Bingxin Chen generated the data, Jingmei Liu and Dean Tian assembled the figure

Figure 2:

Jin Gong generated the data and prepared panel B and C,Yunwu Wang generated the data and prepared panel D and E,Jingmei Liu generated the data and prepared panel A and assembled the figure

Figure 3:

Ping Han generated the data, Jingmei Liu and Dean Tian assembled the figure

Figure 4:

Bingxin Chen and Jin Gong generated the data, Jingmei Liu and Dean Tian assembled the figure

Figure 5:

Bingxin Chen, Yunwu Wang and Jin Gong generated the data, Jingmei Liu and Jiazhi Liao assembled the figure

Figure 6:

Signed for and on behalf of the Author(s):	Print Name:	Date:
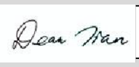	Dean Tian	2018-03-30
